# Bilateral Renal Artery Thrombosis With Distal Abdominal Aortic Dissection as the First Presentation of Takayasu Arteritis

**DOI:** 10.1155/crin/2515985

**Published:** 2026-06-02

**Authors:** Mihiran Thanigasalan, Mehatheepan Rasanayagam, Mathu Selvarajah

**Affiliations:** ^1^ Department of Nephrology, National Hospital of Sri Lanka, Colombo, Sri Lanka, health.gov.lk; ^2^ Department of Radiology, National Hospital of Sri Lanka, Colombo, Sri Lanka, health.gov.lk

**Keywords:** aortic dissection, bilateral renal artery thrombosis, hypertension, large-vessel vasculitis, renal ischemia, Takayasu arteritis

## Abstract

**Background:**

Takayasu arteritis (TAK) is a chronic granulomatous large‐vessel vasculitis that predominantly affects the aorta and its major branches, leading to stenosis, occlusion, or aneurysmal changes. Renal artery disease is common, but acute bilateral renal artery thrombosis causing renal dysfunction is rare.

**Case presentation:**

A 38‐year‐old South Asian male presented with a two‐week history of abrupt‐onset bilateral loin pain and severe azotemia (creatinine 8.25 mg/dL). Inflammatory markers were high (ESR 102 mm/h; CRP 45 mg/L). Ultrasound showed preserved renal size; Doppler suggested globally poor bilateral renal perfusion. CT angiography revealed bilateral renal artery thrombosis, mural thickening of the renal arteries, and a distal abdominal aortic dissection extending to the iliac arteries. Infectious, autoimmune, and limited thrombophilia screens were negative. The clinical and radiological constellation was most consistent with TAK, based on imaging evidence of large‐vessel vasculitis involving bilateral renal arteries and compatible clinical findings. A multidisciplinary team (nephrology, vascular surgery, and rheumatology) advised anticoagulation due to bilateral renal artery thrombosis despite the distal Type B dissection. He received intravenous methylprednisolone followed by oral prednisolone, with recovery of renal function to 1.8 mg/dL and normalization of inflammatory markers in a month.

**Conclusions:**

In young adults presenting with abrupt bilateral renal ischemia and systemic inflammation, TAK should be considered. Early vascular imaging and prompt immunosuppression can preserve renal function. Anticoagulation therapy may be justified in selected cases of thrombosis even in the presence of limited dissection, provided decisions are multidisciplinary and blood pressure is tightly controlled.

## 1. Introduction

Takayasu arteritis (TAK) is a granulomatous vasculitis of the aorta and its primary branches, classically affecting young adults. Renal arteries are frequently involved and may cause renovascular hypertension or ischemic kidney injury, most often via stenosis rather than complete occlusion [1, 2]. TAK presenting with bilateral renal artery thrombosis is rare [3]. The prothrombotic vascular milieu resulting from vascular inflammation likely contributes to the underlying pathophysiology. Aortic dissection is an uncommon but serious complication of active TAK [4, 5]. The superimposed distal aortic dissection in this case may reflect underlying vascular pathology; however, its direct attribution to inflammatory involvement cannot be definitively established in the absence of overt aortic mural thickening.

## 2. Case Presentation

A previously healthy 38‐year‐old South Asian male presented with dull bilateral loin pain and mild nausea for two weeks and reduced urine output for two days. There was no fever, weight loss, claudication, visual symptoms, or arthralgia. He was a nonsmoker. He had taken indigenous medicine for his knee pain for 3 days, a week before the onset of the symptoms.

One week prior to presentation at our center, the patient had sought medical attention at another hospital for the loin pain. He was found to have a serum creatinine of 8.25 mg/dL. Renal replacement therapy was not required. At that time, drug‐induced acute interstitial nephritis was suspected after excluding structural abnormalities, obstruction and infection, and empirical corticosteroid (prednisolone 30 mg daily) therapy was initiated. Renal biopsy was not performed. Despite partial improvement of renal function, he remained symptomatic, and he subsequently developed reduced urine output, prompting referral to our tertiary center for further evaluation (Table 1).

**TABLE 1:** Timeline of events.

Time/Event	Clinical course
2 weeks before admission	Onset of bilateral loin pain and nausea.
1 week before referral	Evaluated at another hospital; creatinine 8.25 mg/dL. Prednisolone 30 mg/day started for presumed acute interstitial nephritis.
Day 0	Referred to tertiary center with persistent symptoms and oliguria. Creatinine 4.56 mg/dL.
Day 2	CT angiography demonstrated bilateral renal artery thrombosis and distal abdominal aortic dissection
Day 3–5	Intravenous methylprednisolone 1 g/day administered.
Day 3	Anticoagulation initiated.
Day 7	Methotrexate initiated as steroid‐sparing therapy
2 weeks	Creatinine improved to 1.9 mg/dL; CRP normalized
4‐week follow‐up	Stable renal function (creatinine 1.8 mg/dL) with imaging evidence of partial recanalization

At our center, blood pressure was 140/90 mmHg bilaterally; normal pulses; no abdominal bruit; no cutaneous lesions or ocular findings. Serum creatinine was 4.56 mg/dL, with normocytic anemia (Hb 10.8 g/dL); electrolytes and coagulation profile were normal. Urinalysis: trace albumin, no hematuria or active sediment. CRP was 45 mg/L; ESR was 102 mm/h. Antinuclear antibody, antineutrophil cytoplasmic antibodies, antiphospholipid antibodies, and D‐dimers were negative. Hepatitis B and C, HIV, syphilis, and tuberculosis screens were negative.

**FIGURE 1:**
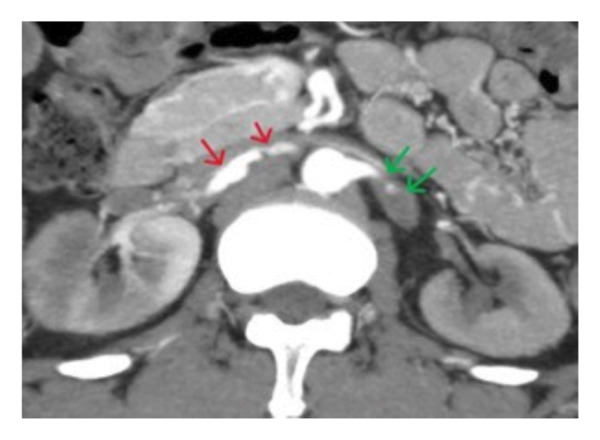
Contrast‐enhanced CT axial image demonstrates bilateral renal artery thrombosis with marked wall thickening of both renal arteries (arrows), suggestive of an underlying inflammatory process. There is significantly reduced flow to both kidneys, resulting in diminished cortical enhancement. The renal cortices appear globally hypo perfused, consistent with acute ischemic injury.

**FIGURE 2:**
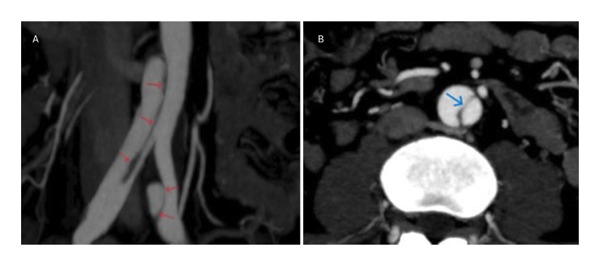
Coronal (A) and axial CT (B) imaging shows an infrarenal aortic dissection that extended distally into both common iliac arteries. The dissection flap (arrow) originated distal to the renal artery origins, with no direct involvement of the renal arteries or their ostia.

**FIGURE 3:**
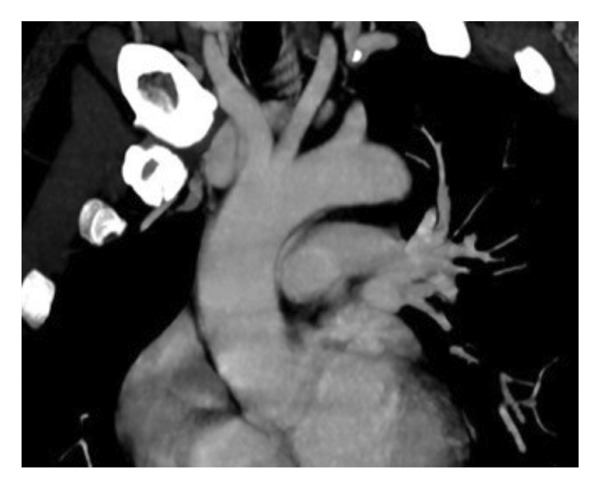
Oblique coronal reformatted image demonstrates that the aortic arch and its major branches appear normal, with no evidence of vasculitic changes, wall thickening, or dissection.

Renal ultrasound showed normal‐sized kidneys with increased echogenicity and preserved corticomedullary differentiation. Renal artery Doppler suggested globally reduced flow bilaterally. CT angiography demonstrated bilateral renal artery thrombosis (Figure 1) with mural thickening and a distal abdominal aortic dissection extending into the iliac arteries (Figure 2). There was no involvement of the aortic arch or its major branches (Figure 3).

The combination of young age, elevated inflammatory markers, and imaging evidence of bilateral renal artery concentric mural thickening was most consistent with the diagnosis of TAK [6]. Differential diagnoses considered included large‐vessel vasculopathy, Behçet disease, IgG4‐related disease, fibromuscular dysplasia, infectious aortitis, and primary thrombophilia; these were rendered unlikely by the imaging pattern and negative laboratory evaluation.

**FIGURE 4:**
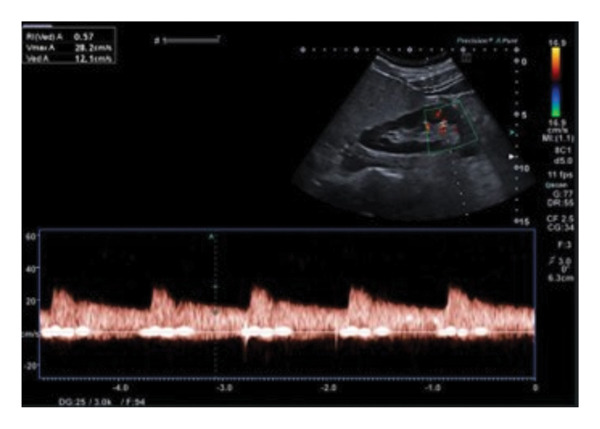
Right renal duplex ultrasound demonstrates improvement in hemodynamic parameters, with relatively low peak systolic velocity (PSV‐ 28.2 cm/s), resistive index (RI‐0.57), and diastolic flow patterns, indicating partial restoration of renal arterial perfusion.

**FIGURE 5:**
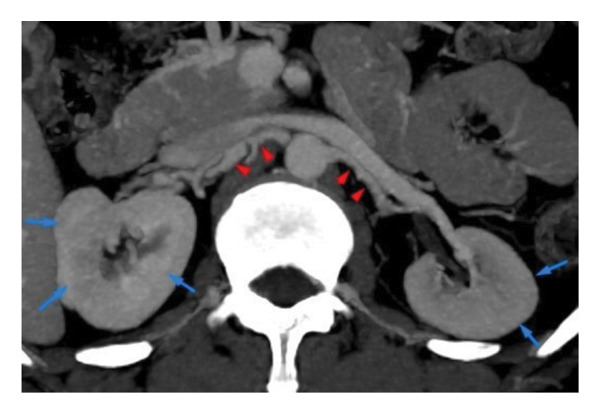
Follow‐up CT shows improved renal arterial flow and cortical enhancement (arrows), with mild residual wall thickening of the bilateral renal arteries (arrow heads), indicating marked resolution of the vascular pathology and restoration of renal perfusion.

The patient received intravenous methylprednisolone 1 g daily for 3 days, then oral prednisolone 1 mg/kg/day with a gradual taper. Methotrexate was initiated early as a steroid‐sparing agent, with a target dose of 15–20 mg weekly, in accordance with recent ACR recommendations favoring early combination therapy in active large‐vessel vasculitis. Anticoagulation was initiated with low‐molecular‐weight heparin (LMWH) and transitioned to warfarin (INR 2–3). Amlodipine and bisoprolol provided blood pressure control. By 2 weeks, creatinine improved to 1.9 mg/dL, and CRP normalized (< 5 mg/L). At 1 month, he remained clinically well with stable kidney function (serum creatinine of 1.8 mg/dL) (Table 1). Surveillance imaging with Doppler ultrasound (Figure 4) and CT aortogram (Figure 5) showed partial recanalization of the renal arteries with adequate blood flow.

## 3. Discussion

Renal artery involvement is common in TAK—nearly half of Asian patients may be affected—typically as stenosis; occlusion/thrombosis is far less frequent [2]. Acute bilateral thrombosis with renal dysfunction, as in this case, is exceptional. The pathophysiology likely reflects intense granulomatous inflammation with endothelial injury and a prothrombotic milieu [1, 2]. Our patient also had distal aortic dissection, which has been reported in association with TAK and requires careful medical management [4] and imaging surveillance [5]. The simultaneous occurrence of arterial thrombosis and aortic dissection presents major therapeutic challenges.

Although there was no involvement of the aortic arch or supra‐aortic branches, the diagnosis of TAK was supported by imaging evidence of large‐vessel vasculitis involving the bilateral renal arteries. It is important to distinguish between classification criteria and clinical diagnosis. The 2022 ACR/EULAR classification criteria were developed primarily for research purposes to standardize patient selection and are not intended to replace clinical judgment in individual cases. This distinction is particularly relevant in cases with isolated branch artery involvement, as observed in this patient.

The presence of concentric mural thickening and symmetric involvement of paired branch arteries represents characteristic imaging findings of inflammatory large‐vessel vasculitis. While abdominal aortic dissection was also present, direct inflammatory involvement of the aortic wall could not be definitively established in the absence of overt mural thickening. Nevertheless, the renal artery findings, in conjunction with systemic inflammation and exclusion of alternative etiologies, strongly supported the diagnosis.

Several alternative diagnoses were considered. IgG4‐related aortitis was unlikely as there was no periaortic soft‐tissue mantle or multiorgan involvement; therefore, serum IgG4 levels and complement levels were not done. Fibromuscular dysplasia was excluded based on the presence of concentric inflammatory mural thickening and thrombosis rather than the classical noninflammatory “string‐of‐beads” appearance. Polyarteritis nodosa was improbable given the absence of microaneurysms, the lack of medium‐sized arterial involvement, and negative hepatitis B serology. Behçet’s disease can present with arterial aneurysms or thrombosis; however, our patient lacked all characteristic mucocutaneous features, including recurrent oral and genital ulcers, eye involvement, or typical dermatologic manifestations. Sarcoid vasculitis was considered unlikely in the absence of pulmonary, lymphatic, ocular, or dermatologic manifestations suggestive of systemic sarcoidosis.

There was no polycythemia or thrombocytosis. There was no prior or family history of deep vein thrombosis. Electrocardiography showed normal sinus rhythm without evidence of atrial fibrillation or ischemic changes. Transthoracic echocardiography demonstrated normal cardiac chambers and valves, with no intracardiac thrombus, vegetation, or cardiac source of embolism. Acquired hypercoagulable states such as antiphospholipid syndrome were ruled out as antiphospholipid antibodies—including lupus anticoagulant, anticardiolipin, and β2‐glycoprotein I antibodies—were negative. Although thrombophilias were considered, extensive testing was not pursued because the patient had isolated arterial involvement without venous thrombosis, which markedly lowers the likelihood of inherited thrombophilic disorders such as protein C/S deficiency, antithrombin deficiency, Factor V Leiden mutation, or prothrombin gene mutation. Moreover, in our resource‐limited setting, such high‐cost investigations are reserved for cases with a higher pretest probability.

Although aortic dissection may cause renal malperfusion through branch compromise, it does not account for concentric mural thickening involving both renal arteries. This vascular pattern is more consistent with an underlying inflammatory arteriopathy affecting primary branch vessels. When interpreted alongside the patient’s inflammatory profile, these findings favored large‐vessel vasculitis over an isolated mechanical vascular process.

High‐dose glucocorticoids remain first‐line for active TAK; steroid‐sparing agents (e.g., methotrexate and azathioprine) or biologics (e.g., tocilizumab) are used for refractory disease or relapse [1, 2]. Tocilizumab was considered; however, given the rapid clinical, biochemical, and radiological response to glucocorticoids and methotrexate, escalation to biologic therapy was not required. Anticoagulation in Type B dissection is typically avoided because of concerns for extension or rupture; however, in this case, multidisciplinary consensus favored anticoagulation [7] given the bilateral renal artery thrombosis with threatened renal viability. The dissection originated distal to the renal artery ostia, with no evidence of malperfusion, expansion, or rupture. Anticoagulation was initiated with strict blood pressure control and close radiological surveillance, including serial CT angiography and renal artery duplex ultrasonography, which confirmed stability of the dissection. He was started on LMWH and then converted to warfarin. Long‐term anticoagulation strategy remains individualized in TAK‐associated arterial thrombosis. In our patient, anticoagulation is planned for at least 6 months with interval reassessment based on vascular imaging, inflammatory activity, and recanalization status.

Although catheter‐directed thrombolysis or endovascular intervention has been successfully reported in selected cases of renal artery thrombosis associated with TAK, these approaches were not pursued in our patient because the concurrent aortic dissection increased procedural complexity and potential vascular risk [3]; however, our case demonstrates that prompt immunosuppression and anticoagulation therapy can achieve recanalization and functional recovery without intervention.

FDG‐PET imaging may have provided additional evidence of active vascular inflammation in this patient, particularly given the absence of classical aortic arch involvement. FDG‐PET can detect metabolically active arterial inflammation even before the development of significant structural vascular changes and may therefore improve diagnostic confidence in atypical presentations of TAK. In addition, FDG‐PET may be valuable during follow‐up for monitoring disease activity, assessing therapeutic response, and identifying subclinical relapse.

The improvement in renal function likely reflects incomplete arterial occlusion with preserved perfusion and early spontaneous or treatment‐related recanalization, preventing irreversible cortical necrosis. Ongoing disease activity and vascular complications contribute to long‐term morbidity, underscoring the need for structured follow‐up with clinical assessment, inflammatory markers, and serial vascular imaging [1].

This case has several limitations. Histopathologic confirmation was not feasible because vascular biopsy was not clinically practical. Advanced vascular imaging modalities such as PET–CT or MRI vessel wall imaging, which may have provided stronger evidence of active vasculitis, were unavailable. In addition, the patient had received corticosteroid therapy prior to referral, which may have partially attenuated inflammatory vascular changes on imaging. Therefore, the diagnosis was based on compatible clinical features, characteristic vascular imaging findings, exclusion of alternative etiologies, and therapeutic response rather than definitive histopathologic confirmation.

## 4. Conclusion

TAK should be included in the differential diagnosis of acute bilateral renal ischemia in young adults, even in the absence of classic constitutional or vascular symptoms. The presence of bilateral renal artery concentric mural thickening provides key imaging evidence supporting inflammatory large‐vessel vasculitis. This case highlights that early vascular imaging, prompt high‐dose glucocorticoid therapy, and a carefully balanced anticoagulation approach can restore renal perfusion and preserve kidney function even when aortic dissection coexists.

To our knowledge, only a few cases of renal artery thrombosis due to TAK have been described, and bilateral involvement with concurrent dissection is exceptionally uncommon. This case contributes valuable evidence that early medical therapy, guided by multidisciplinary collaboration between nephrology, rheumatology, and vascular surgery, can lead to renal recovery without the need for revascularization.

Early recognition, individualized therapy, and structured follow‐up are crucial for optimizing renal and vascular outcomes. Future registry data are needed to guide optimal anticoagulation strategies and long‐term vascular surveillance in such complex vasculitic presentations. Such collaborative evidence will help refine future management algorithms for vasculitic thrombosis involving critical renal vasculature.

## Author Contributions

Mihiran Thanigasalan: conceptualization, data curation, investigation, writing–original draft, and visualization. Mehatheepan Rasanayagam: data curation, investigation, and writing–review and editing. Mathu Selvarajah: supervision, validation, and writing–review and editing.

## Funding

The authors have nothing to report.

## Disclosure

All authors approved the final manuscript.

## Ethics Statement

Ethical approval was not required for this case report according to the policies of the Ethics Review Committee of National Hospital of Sri Lanka.

## Consent

Written informed consent was obtained from the patient for publication of this case report and any accompanying images. A copy of the written consent is available for review by the editor‐in‐chief of this journal.

## Conflicts of Interest

The authors declare no competing interests.

## Data Availability

All data generated or analyzed during this study are included in this article. Further inquiries can be directed to the corresponding author.

## References

[1] Salesi M. and Hosseinpoor S. , Renal Involvement in Takayasu’s Arteritis; a Mini-Review Study, Nickan Research Institute. (2024) 10.34172/jrip.2024.32267.

[2] Li Cavoli G. , Mulè G. , Vallone M. G. , and Caputo F. , Takayasu’s Disease Effects on the Kidneys: Current Perspectives, 2018, Dove Medical Press Ltd, 10.2147/IJNRD.S146355. PMC6101009 30147353

[3] Albaqshi A. , Aljawad M. , Alrasheed S. , Alshaia A. , and Alshehri S. , Thrombosis of Abdominal Aorta and Bilateral Renal Arteries: Endovascular Treatment in Takayasu Disease, Cureus. (2023) 10.7759/cureus.34409. PMC9981228 36874709

[4] Wu X. P. and Zhu P. , Clinical Features of Aortic Dissection Associated with Takayasu’s Arteritis, 2017, Science Press, 10.11909/j.issn.1671-5411.2017.07.010. PMC5545192 28868078

[5] Guo J. , Zhang G. , Tang D. , and Zhang J. , A Case Report of Takayasu Arteritis with Aortic Dissection as Initial Presentation, Medicine. (2017) 96, no. 45, 10.1097/MD.0000000000008610. PMC5690782 29137089

[6] Grayson P. C. , Ponte C. , Suppiah R. et al., American College of Rheumatology/EULAR Classification Criteria for Takayasu Arteritis, Annals of the Rheumatic Diseases. (2022) 81, no. 12, 1654–1660, 10.1136/ard-2022-223482. 36351705

[7] Bocchino P. P. , De Filippo O. , Piroli F. et al., Anticoagulant and Anti-thrombotic Therapy in Acute Type B Aortic Dissection: when real-life Scenarios Face the Shadows of the Evidence-based Medicine, BMC Cardiovascular Disorders. (2020) 20, no. 1, 10.1186/s12872-020-01342-2. PMC6977351 31973746

